# Supplementary material for: Evaluation of the Impact of the Urgent Cancer Care Clinic on Emergency Department Visits, Primary Care Clinician Visits, and Hospitalizations in Winnipeg, Manitoba
Source: Curr Oncol. 2023 Jul 18;30(7):6771–85. doi: 10.3390/curroncol30070496 (PMC10378500; doi:10.3390/curroncol30070496)
Supplement: Supplementary file 1 [file curroncol-30-00496-s001.zip › curroncol-2440256-supplementary.pdf]

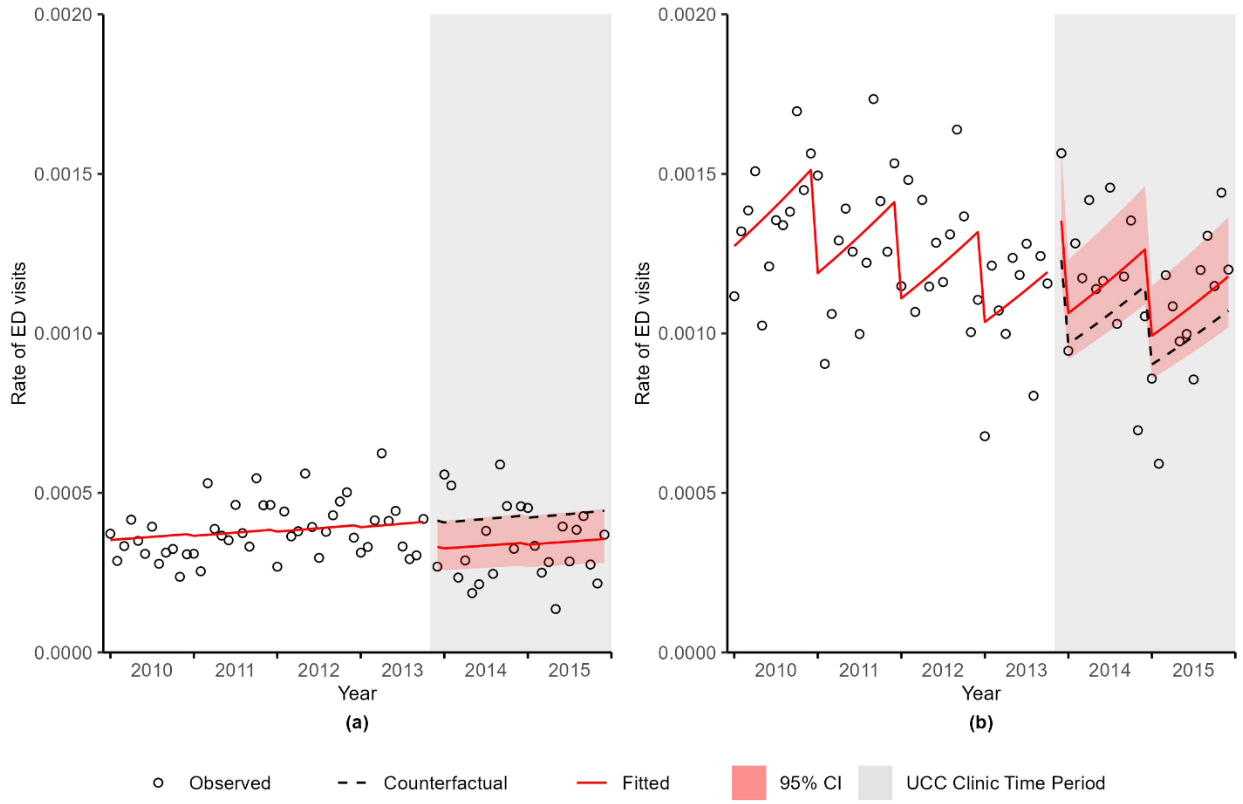

Figure S1. Rate (per person-days) of emergency department visits during the urgent cancer care clinic hours of operation for **(a)** CTAS scores 1 to 2 and **(b)** CTAS scores 3 to 5 by month, Winnipeg, Manitoba.
Abbreviations: CTAS – Canadian Triage and Acuity Scale; ED – emergency department; UCC – urgent cancer care; CI – confidence interval.

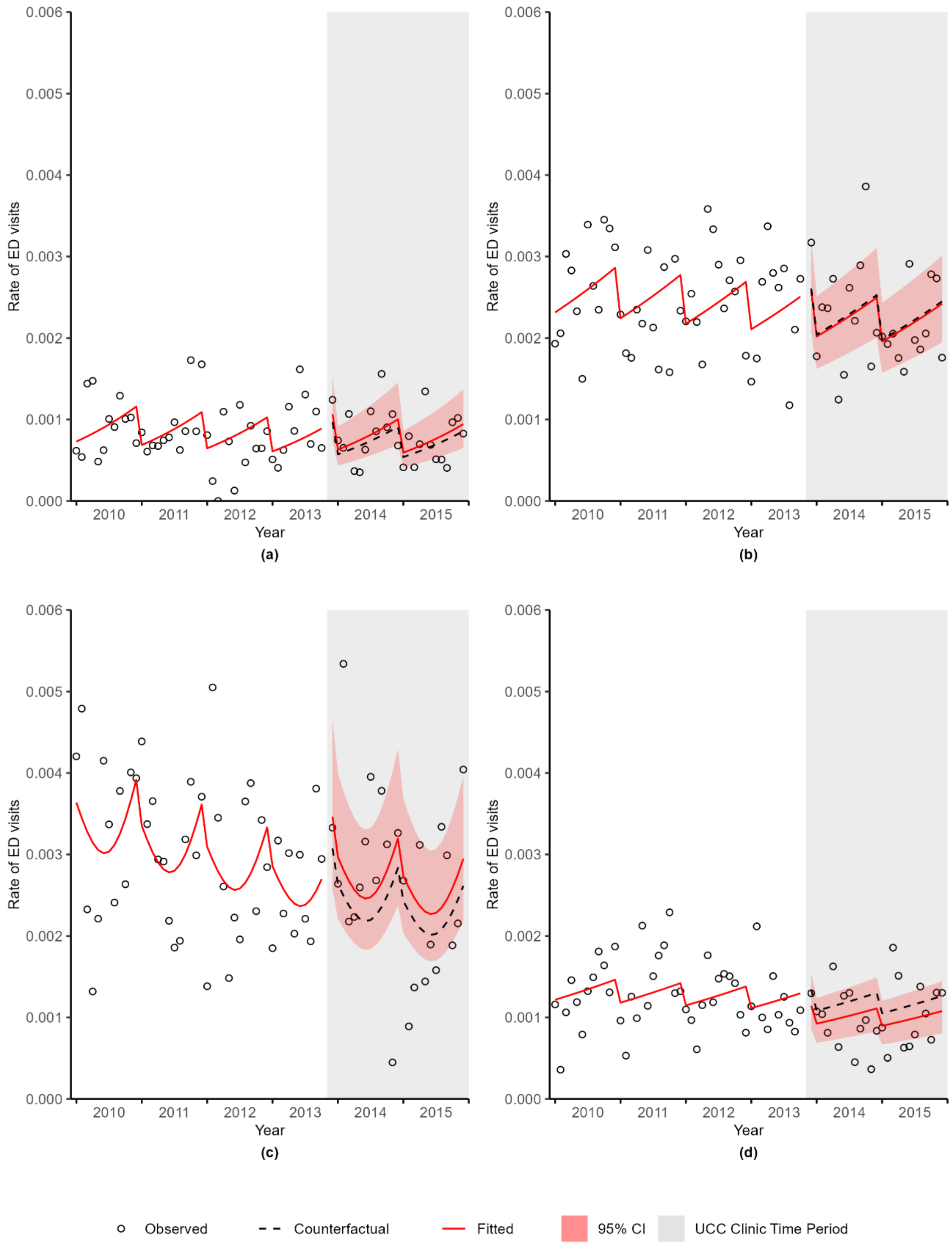

Figure S2. Rate (per person-days) of emergency department visits during the urgent cancer care clinic hours of operation for **(a)** breast cancer, **(b)** digestive cancers, **(c)** lung cancer, and **(d)** genitourinary cancers by month, Winnipeg, Manitoba.

Table S1. Cancer type categories.

Cancer type category	Individual cancer sites
Breast	Breast
Digestive	Esophagus, Stomach, Small Intestine, Colon, Rectal, Anus, Liver, Gallbladder, Pancreas, Other Digestive System
Lung	Lung and bronchus
Genitourinary	Cervix uteri, Corpus uteri, Uterus NOS, Ovary, Other Female Genital System, Prostate, Testis, Penis, Other Male Genital System, Bladder, Kidney and Renal Pelvis, Ureter, Other Urinary System
Hematologic	Hodgkin's Lymphoma, Non-Hodgkin's Lymphoma, Multiple Myeloma, Acute Lymphocytic Leukemia, Chronic Lymphocytic Leukemia, Acute Myeloid Leukemia, Acute monocytic Leukemia, Chronic Myeloid Leukemia, Other Leukemias

Table S2. Ratios and 95% confidence intervals between fitted and counterfactual values.

Outcome	Ratio	95% Confidence Interval	*p*-value
PCC visits	1.06	1.00 - 1.13	0.0389
Hospitalizations	1.07	0.99 - 1.15	0.0737
ED visits	0.96	0.86 - 1.08	0.5053
ED visits during UCC clinic hours	1.03	0.92 - 1.17	0.5778
ED visits at Health Sciences Centre	1.32	1.00 - 1.74	0.0500
ED visits at St. Boniface Hospital	0.69	0.54 - 0.89	0.0041
ED visits at Seven Oaks General Hospital	0.72	0.49 - 1.04	0.0818
ED visits at Grace Hospital	1.05	0.75 - 1.47	0.7719
ED visits at Concordia Hospital	1.29	0.88 - 1.90	0.1913
ED visits CTAS score 1-2	0.80	0.63 – 1.01	0.0641
ED visits CTAS score 3-5	1.10	0.95 – 1.27	0.2007
ED visits for breast cancer	1.10	0.76 - 1.60	0.6103
ED visits for digestive cancers	0.99	0.79 - 1.23	0.9136
ED visits for lung cancer	1.13	0.84 - 1.51	0.4272
ED visits for genitourinary cancers	0.85	0.64 - 1.14	0.2890

Abbreviations: CTAS – Canadian Triage and Acuity Scale; ED – emergency department; UCC – urgent cancer care; PCC – primary care clinician.
